# Sec-CLOCs: Multimodal Back-End Fusion-Based Object Detection Algorithm in Snowy Scenes

**DOI:** 10.3390/s24227401

**Published:** 2024-11-20

**Authors:** Rui Gong, Xiangsuo Fan, Dengsheng Cai, You Lu

**Affiliations:** 1 School of Automation, Guangxi University of Science and Technology, Liuzhou 545006, China; 20230201003@stdmail.gxust.edu.cn (R.G.); 20230203009@stdmail.gxust.edu.cn (Y.L.); 2 Guangxi Collaborative Innovation Centre for Earthmoving Machinery, Guangxi University of Science and Technology, Liuzhou 545006, China; 3 Guangxi LiuGong Machinery Co., Ltd., Liuzhou 545006, China; caids@liugong.com

**Keywords:** DyHead, LIDROR, multimodal object detection, Sec-CLOCs, Wise-IOU, YOLOv8s

## Abstract

LiDAR and cameras, often regarded as the “eyes” of intelligent driving vehicles, are vulnerable to adverse weather conditions like haze, rain, and snow, compromising driving safety. In order to solve this problem and enhance the environmental sensing capability under severe weather conditions, this paper proposes a multimodal back-end fusion object detection method, Sec-CLOCs, which is specifically optimized for vehicle detection under heavy snow. This method achieves object detection by integrating an improved YOLOv8s 2D detector with a SECOND 3D detector. First, the quality of image data is enhanced through the Two-stage Knowledge Learning and Multi-contrastive Regularization (TKLMR) image processing algorithm. Additionally, the DyHead detection head and Wise-IOU loss function are introduced to optimize YOLOv8s and improve 2D detection performance.The LIDROR algorithm preprocesses point cloud data for the SECOND detector, yielding 3D object detection results. The CLOCs back-end fusion algorithm is then employed to merge the 2D and 3D detection outcomes, thereby enhancing overall object detection capabilities. The experimental results show that the Sec-CLOCs algorithm achieves a vehicle detection accuracy of 82.34% in moderate mode (30–100 m) and 81.76% in hard mode (more than 100 m) under heavy snowfall, which demonstrates the algorithm’s high detection performance and robustness.

## 1. Introduction

Most current autonomous driving perception technologies rely on sensors like LiDAR and cameras. However, in snowy conditions, falling snowflakes not only obstruct objects but also diminish their feature information, leading to a poor image quality captured by the camera and posing challenges for image-based object detection algorithms. Furthermore, commercial LiDAR sensors, which typically operate at wavelengths around 900 nm, can detect snowflakes during snowfall. However, they have difficulty in distinguishing between reflections from snowflakes and other objects, causing 3D detectors to mistakenly classify snowflakes and noise as objects. Vehicles are one of the main targets of automated driving systems in adverse weather conditions. Especially in winter environments, vehicles are not only critical dynamic obstacles that affect driving safety and navigation accuracy, but also often become the most difficult to detect and track due to differences in shape and size, as well as snow and complex road conditions. Therefore, accurate vehicle detection is crucial. To this end, this paper proposes a target detection algorithm called Sec-CLOCs, which effectively addresses the challenges of vehicle detection under adverse weather conditions by combining the back-end fusion of camera and LiDAR data.

The key contributions and novel aspects of this research are outlined below:

1. To mitigate the impact of low-quality data on 2D object detection in snowy scenes, we apply the Two-stage Knowledge Learning and Multi-contrast Regularization (TKLMR) algorithm for image preprocessing, enhancing image quality during heavy snowfall. Additionally, the Low-Intensity Dynamic Radius Outlier Removal (LIDROR) filtering algorithm is introduced to reduce the effect of snowflakes on LiDAR point cloud data. Experimental results show that the integration of the TKLMR image snow-removal method and the LIDROR point cloud filtering algorithm significantly reduces the influence of snowflakes on the performance of 2D and 3D object detectors.
2. To enhance the 2D detection algorithm’s performance in snowy environments, we incorporate DyHead as a new detection head built on YOLOv8s, improving the model’s object perception accuracy. Additionally, Wise-IOU is used to reduce reliance on high-quality labeled datasets. Experimental results show that YOLOv8s, combined with DyHead and Wise-IOU, delivers improved accuracy and robustness for 2D detection in snowy conditions.
3. We proposed a back-end fusion algorithm, Sec-CLOCs, based on improved YOLOv8s and SECOND object detection, to further improve vehicle detection performance in snowy environments. Compared to other object detection methods, Sec-CLOCs demonstrated superior results under snowy conditions.

## 2. Related Work

### 2.1. Image Desnowing Algorithm

Snowflakes and snow reduce object features and increase noise in image data, degrading the performance of image-based object detection. Traditional snow-removal algorithms rely on manually constructed filters that identify and separate snowflakes by analyzing the color properties of images (e.g., saturation and sharpness). These methods require the manual identification of snow properties to design appropriate filters for effective snow removal. As an illustration, Pei and co-researchers [1] devised a strategy for detecting and eradicating precipitation, such as rain and snow, from visual data based on the properties of saturation and clarity. Similarly, Wang and team [2] proposed an innovative technique that effectively cleans up rain and snow disturbances within images. Their approach involves breaking down the image into its low- and high-frequency segments, where the fine details of rain and snow are mainly concentrated in the high-frequency domain. The enhancement of the image is achieved through a process that incorporates the detection of rain and snow, coupled with the application of bootstrap filtering techniques, thereby maintaining the vital aspects of the image.

The advent of deep learning has led to a rising trend in the utilization of methods for snow elimination that are grounded in this advanced technology. For instance, DesnowNet [3] utilizes a multi-stage network to sequentially remove both semi-transparent and opaque snow particles. This approach accounts for the semi-transparency and color distortion of snow, enabling more accurate estimation and recovery of details in snow-covered images. When areas are entirely hidden by opaque snow, DesnowNet estimates the residual complement of the snow-free image to restore these occluded details. However, DesnowNet has potential drawbacks, such as producing speckled artifacts when the background is blurred, making it challenging to recover images of snow-covered regions.

Chen et al. [4] introduced a combined process for snow removal that addresses non-transparent snowflakes of varying sizes. However, its ability to generalize is limited, potentially leading to artifacts. Jaw et al. [5] introduced an algorithm for single-image snow elimination that employs a hierarchical dual-tree complex wavelet transformation, further integrating conflict channel loss to improve the effectiveness of snow removal. Similarly, Chen et al. [6] implemented this transform in a network called HDCW-Net for snow information retrieval. Despite its effectiveness, the dual-tree complex wavelet transform approach encounters challenges with dense or translucent snow.

### 2.2. Single-Sensor Object Detection Algorithms

Current algorithms for detecting image objects in severe weather conditions tend to concentrate mainly on rain, fog, and dim lighting scenarios. For example, IA-YOLO [7] integrates a microprocessing image module with CNN-PP to improve detection in fog and low light. TogetherNet [8] combines image restoration and detection tasks for dynamic learning. FogGuard [9] uses YOLOv3 with teacher–student perceptual loss for better foggy detection accuracy, while RDMNet [10] employs a dual-branch network for object detection and image recovery, enhancing performance through multi-scale degradation modeling and feature fusion.

Present LiDAR-based approaches for 3D object detection are broadly categorized into two forms: representation based on voxels and representation based on unprocessed point clouds. In the voxel-based representation, PointPillar [11] transforms point cloud data into columnar feature maps, which are processed using a 2D convolutional network, improving the computational efficiency of 3D object detection. SECOND [12] is optimized for 3D sparse convolution, enabling rapid processing of point cloud data, and introduces a new angle coding scheme and data enhancement method, allowing it to handle large object detection with excellent performance while maintaining fast processing speeds. In the raw point cloud-based representation, PointNet [13] is a pioneering method for object detection that addresses the challenges of traditional convolutional neural networks, which struggle to perform feature extraction on raw point clouds due to their disorder and irregularity. PV-RCNN [14] combines the advantages of both voxelized representation and raw point cloud representation in a two-stage network, thereby improving 3D object detection performance while managing memory consumption effectively.

### 2.3. Multi-Sensor Fusion Algorithms

In the field of object detection, multimodal fusion methods enhance detection by merging data from different sensors. These methods are usually categorized into three fusion stages: early fusion, deep fusion and late fusion.

Early fusion, or data layer fusion, merges raw point cloud and image data to leverage cross-modal interactions. Although effective, it faces challenges like data alignment and computational demands. MVX-Net [15] is an example of this approach, proposing fusion strategies like PointFusion, which combines each 3D point with RGB image features using pre-trained 2D networks for simultaneous learning. Point Augmenting [16] improves 3D object detection through cross-modal data augmentation by decorrelating point clouds with CNN features and incorporating virtual objects into both imagery and point cloud data throughout the training process.

Deep fusion combines LiDAR point clouds and image data, requiring precise alignment and complex networks. For example, STD [17] proposed a two-phase approach that brings in the notion of spherical reference points, facilitating the more precise prediction of the position and dimensions of objects in its initial stage. It also includes a PointsPool Layer for extracting features from candidate regions in the second stage. EPNet [18] enhances point features with semantic image features without requiring any image annotation, using a consistent enforced loss to address localization and classification consistency. 3D-CVF [19] implements a cross-view feature fusion technique, converting 2D camera data into feature maps that align closely with 3D LiDAR information in a bird’s-eye view (BEV). It achieves this through an automatic projection calibration process. A gated feature fusion network is then applied to blend these features, determining the region’s significance based on the relative importance of camera and LiDAR data for optimal fusion.

Late fusion systems process data from different modalities separately before fusing them at the decision level, which has many significant advantages. This flexibility allows the system to adapt to changing application scenarios. If there is a problem or loss of data in one modality, it will not affect the processing of other modalities, thereby improving the robustness of the system. For instance, CLOCs [20] can be used with any pre-trained 2D and 3D detector without the need for additional training. This method exploits geometric and semantic consistency and fuses the detection results of different modalities through a probability-driven learning-based method. This leads to a considerable advancement in the efficiency of object detection.

## 3. Methods

**Figure 1:**
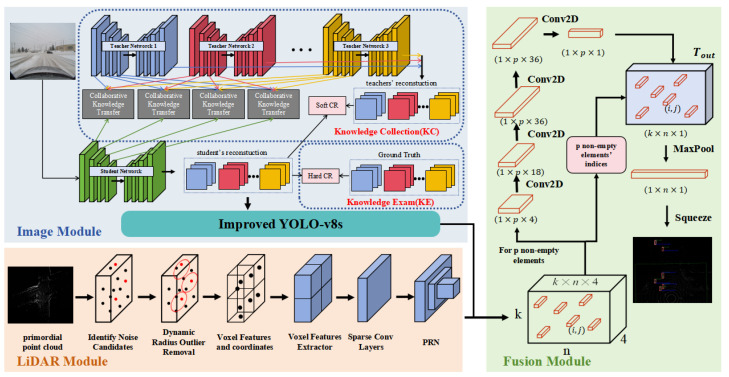
The whole process of Sec-CLOCs, an object detection algorithm based on the back-end fusion of a camera and LiDAR.

In this study, we propose the Sec-CLOCs back-end fusion object detection algorithm to address the challenge of detecting vehicles in snowy environments. To address the image distortion problem in such conditions, we introduce an integrated severe weather removal technique based on two-stage knowledge learning, which utilizes a unified framework with pre-trained weights to efficiently address the problem. In the image detection section, we improve the sensing accuracy by merging DyHead into the detection architecture of YOLOv8s and utilize Wise-IOU to reduce the dependence on the quality of premium datasets. In the area of point cloud detection, we employ the Low-intensity Dynamic Radius Outlier Removal (LIDROR) method to mitigate the impact of snowflakes on the raw point cloud data, which is then complemented by the SECOND [12] method to recognize 3D objects. In the subsequent fusion phase, we utilize the CLOCs [20] post-fusion algorithm to merge the detection data from two different sensors. Due to the versatility and modular design of the algorithm, we can train the 2D and 3D detectors separately, which greatly improves the training efficiency of the system. Figure 1 illustrates the whole flow of Sec-CLOCs, the vehicle detection algorithm based on a camera and LiDAR back-end fusion proposed in this paper.

### 3.1. Image Snow-Removal Algorithm

To enhance image quality during snowy conditions, this paper employs an integrated severe weather removal method based on two-stage knowledge learning [21]. This method consists of two phases:

Knowledge Collation (KC) Phase: In this phase, the student network learns to handle various weather conditions by collaborating with multiple teacher networks, each trained for a specific recovery task. The student network integrates knowledge from these teachers using Collaborative Knowledge Transfer (CKT). Characteristics extracted from the teacher and student networks are mapped to a shared feature domain through a Progressive Feature Projection (PFP) module. Subsequently, Bidirectional Feature Matching (BFM) is executed to promote effective knowledge dissemination. The PFP is a learnable module that determines the optimal feature space for common feature learning. BFM constrains the learned features by re-projecting the teacher networks’ features back into the original input space and measuring their differences from the original features.

**Figure 2:**
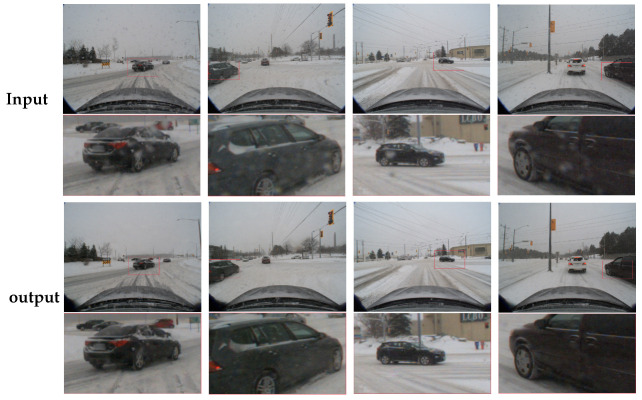
TKLMR snowflake removal results. Input is the input raw image and Output is the result after applying the TKLMR severe weather removal algorithm.

Knowledge Examination (KE) Phase: After training in the KC phase, the student network is ready to handle diverse weather types without relying on the teacher networks. In this phase, the network’s robustness and discrimination are enhanced through more stringent constraints. The aim is to improve the overall weather recovery performance by applying stricter regularization, using the Multi-Contrast Regularization (MCR) loss. This loss function comprises Soft Contrast Regularization (SCR) and Hard Contrast Regularization (HCR). SCR, used in the KC phase, reduces learning difficulty by leveraging the teacher networks’ predictions as positive samples. In the KE phase, HCR enhances performance across multiple weather types by utilizing the actual ground truth of input images as positive samples and the degraded images across all weather conditions as negative samples, thereby improving the network’s discrimination ability. The effect is shown in Figure 2.

### 3.2. Improved 2D Detector

This study aims to boost the efficacy of 2D image detection in snowy conditions by altering YOLOv8s. The conventional detection head of YOLOv8s is substituted with DyHead (Dynamic Head) [22], and Wise-IOU [23] is employed as a replacement for the original loss function to reduce the negative impact of subpar sample quality. DyHead notably enhances the model’s object detection head’s perceptual abilities without incurring extra computational expenses. It achieves scale perception by incorporating an attention mechanism across feature layers, spatial perception through an attention mechanism at different spatial positions, and task perception by employing an attention mechanism in the output channel.

Specifically, given the feature tensor $F\in R^{L\times S\times C}$, the general formula for applying self-attention is as follows:

(1) W(F)=π(F)·F

where π(·) denotes an attention mechanism. A simplified method for deploying this mechanism involves using a fully connected layer. Nevertheless, due to the tensor’s elevated dimensionality, learning the attention mechanism across its entire scope is computationally demanding and frequently infeasible. Consequently, the attention mechanism is decomposed into three consecutive attentions, with each attention focused solely on one aspect:

(2) $$W\left(F\right)=\pi_{C}\left(\pi_{S}\left(\pi_{L}\left(F\right)\cdot F\right)\cdot F\right)\cdot F.$$

The functions $\pi_{L(\cdot )}$, $\pi_{S(\cdot )}$, and $\pi_{C(\cdot )}$ signify three separate attention mechanisms, each applied to the dimensions L, S, and C, respectively.

The scale-sensitive attention mechanism, $\pi_{L(\cdot )}$, adaptively merges features from various scales according to their semantic relevance.

(3) $$\pi_{L}\left(F\right)\cdot F=\sigma \left(f\left(\frac{1}{SC}\sum_{S,C}F\right)\right)\cdot F$$

where $\begin{array}{c} f(\cdot ) \end{array}$ is a linear function approximated by a 1×1 convolutional layer, while σ(x)=max(0,min(1,x+12)) constitutes a hard sigmoid activation.

Spatial Perception Attention $\pi_{S}$ is a module designed for spatial perception based on fused features. Due to the complexity of dimension S, the module is split into a two-phase process: initially, attention learning is made sparse via deformable convolution, followed by the consolidation of features across identical spatial coordinates within different layers:

(4) $$\pi_{S}\left(F\right)\cdot F=\frac{1}{L}\sum_{l=1}^{L}\sum_{k=1}^{K}w_{l,k}\cdot F\left(l;p_{k}+\Delta p_{k};c\right)\cdot \Delta m_{k}$$

In this context, K denotes the count of sparsely sampled locations, with pk+Δpk representing the positional shift determined by the spatial offset Δpk that targets the distinctive area, and Δmk signifies the self-learned importance scalar at location pk. These values are derived from the input characteristics at the intermediate stage of F.

The task-specific attention $\pi_{C}$ adaptively toggles the activation and deactivation of feature channels to cater to various tasks:

(5) $$\pi_{C}\left(F\right)\cdot F=max\left(\alpha^{1}\left(F\right)\cdot F_{c}+\beta^{1}\left(F\right),\alpha^{2}\left(F\right)\cdot F_{c}+\beta^{2}\left(F\right)\right)$$

**Figure 3:**
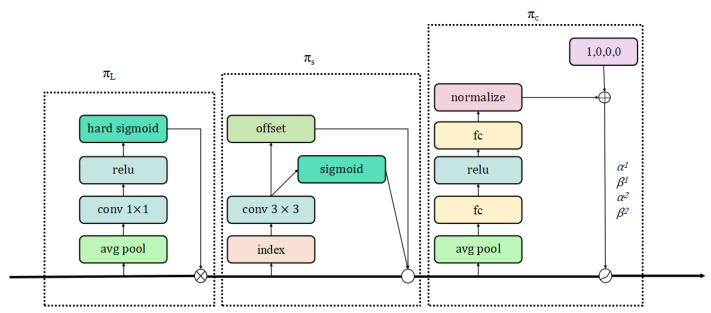
Detailed configuration of each attention module of the DyHead block.

**Figure 4:**
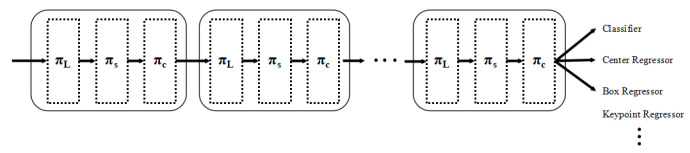
DyHead single-stage detection configuration.

**Figure 5:**
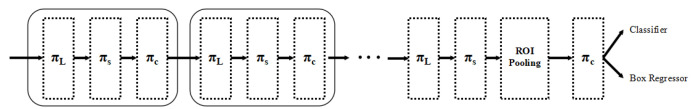
DyHead two-stage detection configuration.

In this context, Fc is the feature slice of the cth channel, with ${\left[\alpha^{1},\alpha^{2},\beta^{1},\beta^{2}\right]}^{T}=\theta \left(\cdot \right)$ serving as a hyperfunction that learns to govern the activation threshold. These equations can be recursively nested, allowing for the effective stack of multiple $\pi_{L}$, $\pi_{S}$, and $\pi_{C}$ modules. The detailed configuration of the DyHead module is shown in Figure 3. Figure 4 is for a single-stage inspection configuration, and Figure 5 is for a two-stage inspection configuration.

In the snowy autonomous driving dataset, falling snowflakes and light may cause poor annotation of the dataset, resulting in a large IoU loss for the 2D detector when detecting quality anchor boxes. As a result, we replaced the standard loss function in YOLOv8s with the Wise-IOU method. Wise-IOU represents a novel approach to bounding box regression, utilizing a flexible, non-monotonic focusing strategy to gauge the efficacy of anchor boxes, moving away from the traditional IoU evaluation approach. This mechanism determines anchor box quality based on “outliers” rather than relying exclusively on IoU values. In addition, the Wise-IOU uses an intelligent gradient gain strategy to focus on high-quality anchor frames and mitigate the effects of low-quality samples, ultimately focusing on improving the optimization of medium-quality anchor frames and improving the effectiveness of the detection frame. Specifically, Wise-IOU is defined as follows:

(6) $$L_{WloUv3}=rR_{WloU}L_{IoU}xc$$

(7) $$R_{WloU}=\exp\left(\frac{{\left(x-x_{gt}\right)}^{2}+{\left(y-y_{gt}\right)}^{2}}{{\left(W_{g}^{2}+H_{g}^{2}\right)}^{*}}\right)$$

(8) $$r^{2}=\frac{\beta }{\delta \alpha^{\beta -\delta }}$$

where $W_{g}$ signifies the breadth of the minimum outer bounding box, while $H_{g}$ signifies the height of the minimum outer bounding box and $W_{g}^{2}+H_{g}^{2}$ signifies the diagonal length of the minimum outer bounding box. The symbol * indicates a separation operation to forestall the generation of gradients by the $R_{WloU}$ that could impede convergence, where C is a constant value and δ makes r = 1 when β=δ.

### 3.3. Three-Dimensional Object Detection

This paper utilizes SECOND [12] as a 3D object detector. SECOND features a sparse convolutional network architecture that enables the model to effectively detect objects of different shapes and sizes while ensuring high efficiency. Additionally, SECOND also introduces an enhanced angle loss function to optimize orientation estimation performance and implement a novel data augmentation technique to boost the model’s generalization capability and convergence speed.

To address the issue of noise, such as snowflakes, affecting data collected by LiDAR in snowy environments, we introduced the LIDROR algorithm for point cloud filtering, which is grounded in the SECOND architecture.

**Figure 6:**
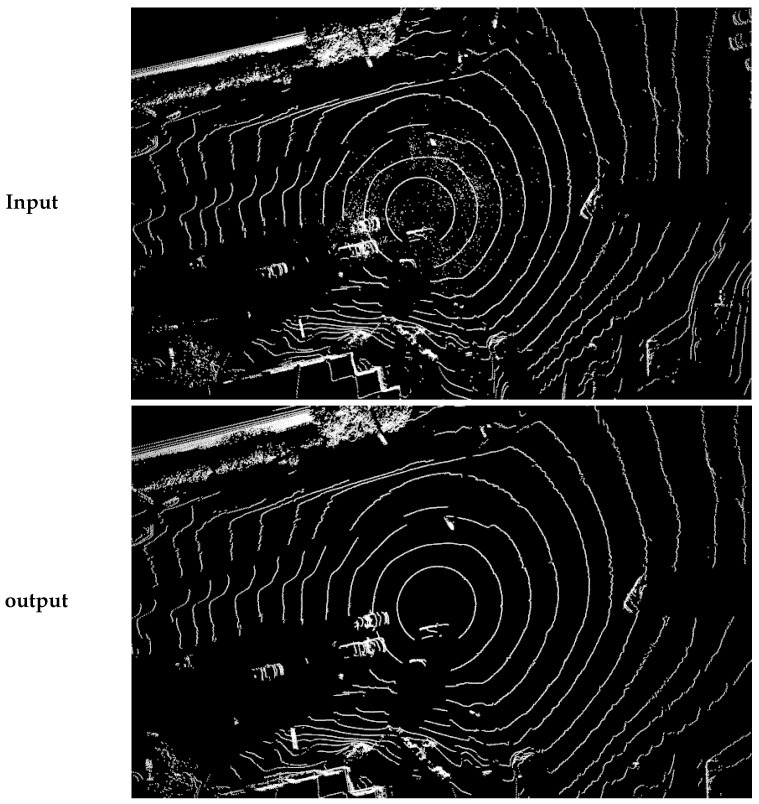
LIDROR processing results. The input is the original point cloud visualization and the output is the point cloud visualization after LIDROR point cloud filtering.

In [24], taking advantage of the fact that snow particles exhibit lower intensity values compared to other objects, a LIOR filtering algorithm was proposed that utilizes intensity data from LiDAR 3D point clouds to remove snow. This filtering process consists of two stages: initially, it scrutinizes each point to mark the point where the intensity reading falls below a predetermined threshold, e. Subsequently, ROR filtering is performed on potential outliers pinpointed during the initial scan, and the points designated as outliers are removed from the point cloud dataset. A notable characteristic of the LIOR filtering process is its selective application of the ROR filter to certain points, which enables the LIOR method to operate at enhanced speeds in contrast to the DROR filter [25], yet it preserves similar efficacy in eliminating snow particles [24]. However, the traditional LIOR filter struggles with variations in distance, prompting the introduction of a new filter, LIDROR [26]. Specifically, during the LIOR filter’s second phase, the ROR filter is substituted with the DROR filter, which utilizes a variable search radius to overcome the limitations of the ROR filter. The parameters for dust extraction, including the fixed multiplier Φ and the minimum acceptable number of points within the search area, necessitate optimization. These settings are fine-tuned in response to their influence on the filter’s effectiveness and robustness across varying dust conditions. Furthermore, the LIDROR filter permits the adjustment of a higher intensity threshold without sacrificing crucial non-dust data, thereby optimizing dust extraction. As depicted in Figure 6, the LIDROR filter successfully diminishes snowflake interference in the point cloud dataset.

### 3.4. Multi-Sensor Fusion Detection Module

This paper adopts the CLOCs object detection algorithm as the fusion algorithm for 2D and 3D detection results. The algorithm first uses the camera and LiDAR independently for 2D and 3D object detection to generate two sets of candidate objects, and then calculates the geometric consistency (through IoU) and semantic consistency (through category matching) between these candidate objects to construct a sparse tensor containing these scores. Next, a 2D convolutional neural network is used to process the non-zero elements in this sparse tensor to fuse information from different modalities. The merged characteristics are then translated into the ultimate detection outcomes using a maximum pooling operation, resulting in precise and efficient 3D object detection.

When performing object detection in an image, the 2D detection technology generates a set of two-dimensional bounding boxes and corresponding confidence levels. For n potential objects in an image, the 2D detection results can be described by the following expression:

(9) $$\begin{array}{cc} & B^{2D}=\left\{B_{1}^{2D},B_{2}^{2D},\ldots B_{n}^{2D}\right\},\\ & B_{k}^{2D}=\left\{\left[x_{k1},y_{k1},x_{k2},y_{k2}\right],s_{k}^{2D}\right\} \end{array}$$

$B^{2D}$ denotes the collection of n candidate frames in an image. For the kth 2D detection candidate among these frames, $x_{k1},y_{k1}$ denotes the pixel location of the top-left boundary of the frame, $x_{k2},y_{k2}$ marks the pixel location of the bottom-right boundary of the frame, and $s_{k}^{2D}$ conveys the confidence level associated with the detection.

In the object detection scenario of a 3D point cloud, the output includes the 3D dimensions, spatial position and rotation angle of each candidate object. For k 3D detection candidates in the point cloud, the 3D detection results can be described by the following expression:

(10) $$\begin{array}{cc} & B^{3D}=\left\{B_{1}^{3D},B_{2}^{3D},\cdots B_{n}^{3D}\right\},\\ & B_{k}^{3D}=\left\{\left[h_{k},w_{k},l_{k},x_{k},y_{k},z_{k},\theta_{k}\right],s_{k}^{3D}\right\} \end{array}$$

$B^{3D}$ represents the collection of n 3D potential frames within a point cloud, where the kth potential 3D detection frame is indicated by $B_{k}^{3D}$, and $\left[h_{k},w_{k},l_{k},x_{k},y_{k},z_{k},\theta_{k}\right]$ encapsulate the 7 attributes of the candidate frame in the point cloud. $s_{k}^{3D}$ are the confidence scores of the represented point cloud candidate frames.

We use a new fusion network structure to re-evaluate all candidate frames. For the k results of 2D detection and n results of 3D detection, we synthesize the representation by creating a tensor T of k×n×4:

(11) $$T_{i,j}=\left\{IoU_{i,j},s_{i}^{2D},s_{j}^{3D},d_{j}\right\}$$

where $IoU_{i,j}$ represents the geometric consistency between the i-th detection result in the image and the j-th in the point cloud. $s_{i}^{2D}$ is the confidence score of the i-th detected object in the two-dimensional detection and $s_{j}^{3D}$ is the confidence score in the point cloud scene. $d_{j}$ represents the normalized distance from the j-th object detected in the point cloud scene to the ground. In this way, the result can be represented as a four-dimensional tensor of coefficients, which can be directly input into the convolutional network for fusion. Elements $T_{i,j}$ with zero IOU will be eliminated because they are geometrically inconsistent.

## 4. Experiment Preparation

### 4.1. Experimental Dataset

The Canadian Adverse Driving Conditions (CADCs) [27] Dataset is the world’s first dataset customized for the study of self-driving vehicles in cold environments. The dataset collects data from a variety of winter driving scenarios during the winter months in the Waterloo Region, Canada, and consists of 56,000 images and 7000 LiDAR scans covering 75 unique scenarios with frame counts ranging from 50 to 100 per scenario. The dataset is carefully labeled with 10 object classes, including vehicles, pedestrians, trucks, etc., and is equipped with an extensive sensor array consisting of one LiDAR, eight cameras, and GPS/IMU data post-processing. Vehicles are the most critical targets for automated driving systems in snowy weather conditions, and they are the main dynamic obstacles affecting safety and navigation in winter environments. Especially on snow-covered roads, the visibility of moving vehicles is severely affected; at the same time, stationary vehicles (e.g., parked vehicles) are usually more difficult to detect and recognize due to snow accumulation, reduced visibility, and varying vehicle sizes and shapes. Therefore, vehicles are the most challenging and relevant target category in our study, and we specifically chose a sample containing both stationary and moving vehicles as the dataset for this study. In this paper, 5250 samples of camera and LiDAR data collected in snowy environments are used. To ensure the robustness and generalizability of the data, these samples cover a wide range of driving environments, including city streets, highways, and country roads, to fully reflect the diversity of driving in snow. Each driving environment includes snow-covered roads, snow on vehicles, and reduced visibility due to snowfall, ensuring that the dataset effectively captures the unique challenges vehicles face in winter driving scenarios. All 5250 samples were divided into a training, validation, and test set in a 6:2:2 ratio, with the training set containing 3150 samples for model training, and the validation and test sets each containing 1050 samples to ensure that model performance evaluations are based on independent datasets.

### 4.2. Parameter Settings

Some training parameters of YOLOv8s are shown in Table 1, using the open hyperparameters set in the code.

Some training parameters for CLOCs are provided in Table 2, using the open hyperparameters set in the code.

## 5. Experimental Results

### 5.1. YOLOv8s Experiment

In this study, Precision, Recall, mAP 50, mAP 50-95, and F1 score are used as evaluation metrics for YOLOv8s. Precision measures how many of the samples predicted by the model to be positive are actually true positive samples. Recall measures the proportion of all true positive samples that are successfully predicted by the model. The mAP 50 is the Average Precision (AP) of the model at a threshold of IoU = 0.50. The mAP 50-95 is a more rigorous calculation of mAP, which calculates the average Precision at multiple IoU thresholds from IoU = 0.50 to IoU = 0.95 in steps of 0.05. F1 score is the reconciled average of Precision and Recall and is designed to measure the overall performance of the model. It combines the accuracy and Recall of the model, especially if the dataset is not balanced in terms of categories. The F1 score is a better reflection of the model’s performance.

This paper adds different modules to YOLOv8s for comparative experiments, and the results are shown in Table 3. In heavy snow weather, the annotation effect of the dataset will be affected, resulting in a large IoU loss when the 2D detector detects low-quality anchor boxes. Wise-IoU mainly focuses on optimizing medium-quality anchor boxes and improving the effectiveness of detection boxes, thereby significantly improving the detection performance of 2D detectors under severe weather conditions. In addition, compared with other modules, DyHead unifies different target detection heads by introducing an attention mechanism, thereby significantly improving the model’s expression ability on the target detection head without increasing the computational burden. Table 3 shows that YOLOv8s+DyHead and YOLOv8s+Wise-IoU perform better than other models under snowy conditions. More importantly, the combination of these two modules fully utilizes their complementary advantages and significantly improves the overall performance of YOLOv8s, especially in terms of detection accuracy and F1 score.

**Figure 7:**
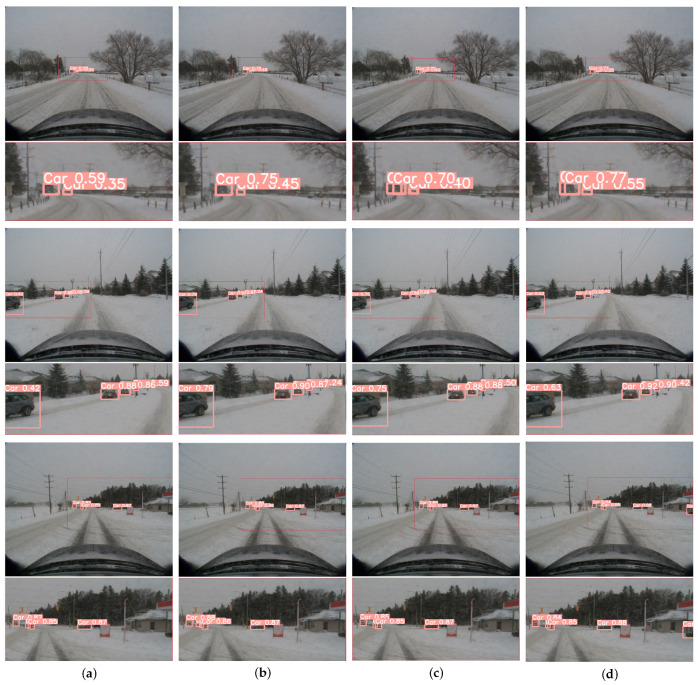
Improved YOLOv8s ablation experiment visualization results. (**a**) Original YOLOv8s detection result. (**b**) YOLOv8s+DyHead detection result. (**c**) YOLOv8s+Wise-IOU detection result. (**d**) YOLOv8s+DyHead+Wise-IOU detection result. (We suggest viewing in enlarged mode).

Through the fusion of YOLOv8s with DyHead, the model achieved a 0.72% boost in mean Average Precision (mAP) over the conventional YOLOv8s. DyHead streamlines scale, spatial, and task awareness by employing attentional processes across different feature hierarchies, spatial locations, and channel outputs, which sharpens the model’s responsiveness to a range of object sizes without incurring additional computational costs, notably improving vehicle detection performance. Next, replacing YOLOv8s’ loss function with Wise-IOU led to the model’s mAP increase of 2.63% on the CADC dataset. The results show that Wise-IOU reduces the IoU loss generated when generating high-quality anchor boxes due to the poor annotation quality of the dataset, making the model more adaptable to the snowy environment. Finally, after combining DyHead and Wise-IOU, it was found that they did not interfere with each other, allowing the model to achieve the highest level of mAP value on the CADC dataset, which is 2.99% higher than the ordinary YOLOv8s.Experiments show that the improved YOLOv8s improves the accuracy of image object detection compared with the original YOLOv8s. The visualization results of YOLOv8s, YOLOv8s+dyhead, YOLOv8s+Wise-IOU and YOLOv8s+dyhead+Wise-IOU are shown in Figure 7.

### 5.2. Sec-CLOCs Ablation Experiment

This study uses the evaluation criteria of the KITTI dataset [28,29], classifying objects into three categories: easy (within 30 m), moderate (30–100 m), and hard (beyond 100 m). The Average Precision (AP) of bird’s eye view ($A_{\mathrm{BEV}}$) and 3D detection ($A_{3D}$) was tested at an IoU threshold of 0.7, following standards for vehicle categories and previous research.

In order to evaluate the overall performance improvement, ablation experiments were conducted to compare the improved Sec-CLOCs algorithm with the original CLOCs algorithm; the results are shown in Table 4. First, the LIDROR point cloud filtering algorithm is introduced into the original CLOCs algorithm. In the easy mode, $A_{\mathrm{BEV}}$ is improved by 4.01%, and $A_{3D}$ is improved by 10.01%. The LIDROR point cloud filtering algorithm efficiently removes the mid-range and far-range snowflake noise in the LiDAR raw data, which significantly reduces the interference of snowflakes on the 3D detection results, reduces the possible pseudo-targets, and thus improves 3D detection and BEV boundary detection. The accuracy of 3D detection and the BEV bounding box at medium and long ranges is improved.

Subsequently, in the easy mode, $A_{\mathrm{BEV}}$ and $A_{3D}$ are improved by 6.74% and 28.70%, respectively, when combined with the improved YOLOv8s 2D image detection results. By replacing the original loss function of YOLOv8s with Wise-IOU, the accuracy of the 2D detector for object localization and bounding box detection at medium and long distances is further improved, which reduces the impact of the poor quality of data annotation in snowy weather. Thanks to the dynamic design of the DyHead, the detector head is better able to cope with different sizes, shapes, and types of targets, allowing the improved YOLOv8s to achieve significant gains in medium- and long-range vehicle detection. In moderate mode, $A_{\mathrm{BEV}}$ and $A_{3D}$ show 21.67% and 47.65% improvements over the baseline, respectively, and in hard mode, $A_{\mathrm{BEV}}$ and $A_{3D}$ show 18.26% and 38.51% improvements, respectively.

Finally, the introduction of the TKLMR image processing method effectively mitigates the negative impact of snowflakes on the camera image and further improves the performance of the 2D detector. In the easy mode, $A_{\mathrm{BEV}}$ improves 11.58% and $A_{3D}$ improves 43.18% compared to the benchmark; in the moderate mode, $A_{\mathrm{BEV}}$ and $A_{3D}$ improve by 23.33% and 48.85%, respectively. In hard mode, $A_{\mathrm{BEV}}$ and $A_{3D}$ improved by 19.69% and 39.96%, respectively.

### 5.3. Sec-CLOCs Comparison Experiment

Furthermore, this study conducts a comparative analysis of the Sec-CLOCs method against the AVOD [30], F-PointNet [31], TANet [32], PointRCNN [33] and Snow-CLOCs [34] approaches using an identical dataset. The findings are detailed in Table 5.

Among these evaluation metrics, Sec-CLOCs demonstrated significant advantages for medium- and long-range vehicle target detection under heavy snowfall. Specifically, by adopting the improved YOLOv8s as the 2D detector, the detection performance of different sizes, shapes and types of vehicle targets in snowy environments is significantly improved and further optimized for medium- and long-range vehicle targets. Meanwhile, with the help of the LIDROR point cloud filtering algorithm, the interference of snowflakes on the 3D detector in moderate and heavy modes is significantly reduced. Under the condition of an IoU threshold of 0.7, the bird’s eye view detection accuracies of Sec-CLOCs in medium and hard modes are 82.34% and 81.76%, respectively, and the 3D detection frame accuracies are 64.69% and 63.78%, respectively. By fusing the 2D and 3D detection results, Sec-CLOCs further improves the efficiency of utilizing the information of multiple viewpoints, which means the detection accuracy in the complex environments is significantly improved. This kind of back-end fusion technology (CLOCs) can effectively integrate data from different sensors, overcoming the problem of information loss or error accumulation that a single sensor may face, especially in the detection of medium- and long-range targets, which shows a strong advantage. The TKLMR image processing method used in this paper is a removal method for multiple severe weather conditions, which may not be as effective as those algorithms optimized for specific weather conditions (e.g., heavy snow, haze, etc.) in removing multiple weather disturbances when compared to image processing algorithms optimized for a single weather condition, and thus the targets in the images may not be clear enough. In addition, the improvements to YOLOv8s in this paper focus on optimizing the detection performance of small targets at medium and long distances, especially in more complex or difficult environments (e.g., heavy snowfall or challenging 3D environments), but these improvements also introduce a certain amount of computational overhead or additional complexity, which does not result in a significant increase in performance in the relatively simple conditions in the easy mode. Compared to Snow-CLOCs, the architecture of Sec-CLOCs focuses more on the detection of medium- and long-range vehicle targets in snowy environments and achieves significant improvements in this regard.

**Figure 8:**
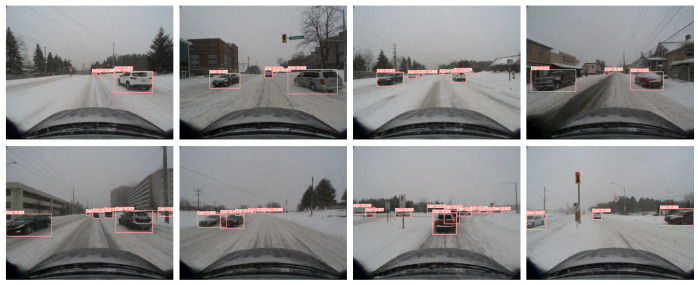
Results of 2D object detection on the validation set of the CADC public dataset. The red box indicates the object position predicted by our algorithm, and the corresponding number indicates the confidence score.

**Figure 9:**
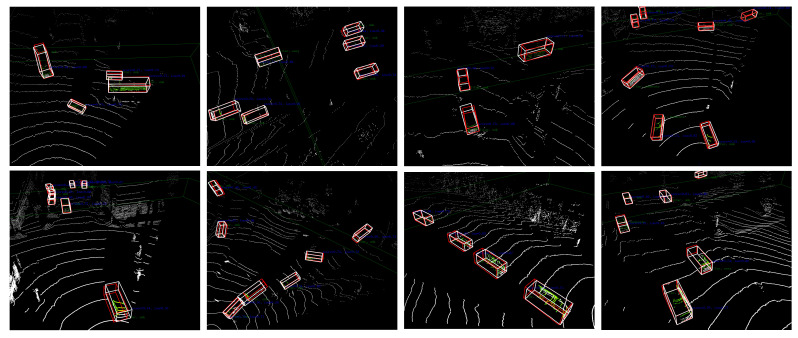
Results of 3D object detection results on the validation set of the CADC public dataset. The red box indicates the object position predicted by our algorithm, while the white box indicates the ground truth object position.

As shown in Figure 8 and Figure 9, the 2D detection performance of Sec-CLOCs and the 3D detection performance of the final fused detection are demonstrated, proving that it achieves better 2D and 3D detection performance in snowy weather.

## 6. Conclusions

This paper proposes an object detection method, Sec-CLOCs, in snowy environments based on the back-end fusion of camera data and LiDAR. This algorithm is divided into 2D detection part, 3D detection parts and fusion parts. For the 2D detection part, the images collected in snowy weather are preprocessed using Two-stage Knowledge Learning and the Multi-contrastive Regularization (TKLMR) bad weather removal algorithm. At the same time, the YOLOv8s algorithm is improved, and DyHead is introduced to replace the original detection head and combined with Wise-IOU. For the 3D detection part, the point cloud data collected in snowy weather are preprocessed using the LIDROR point cloud filtering algorithm, and then SECOND is used for 3D object detection. For the fusion part, the output results of the 2D detector and 3D detector are fused using the CLOCs back-end fusion algorithm. The experimental results show that the algorithm proposed in this paper achieves 84.50%, 82.34% and 81.76% vehicle detection accuracy in easy, moderate and hard modes, respectively, and achieves good detection performance in snowy environments. Currently, the method is designed for vehicle detection and is optimized for snowy conditions. This limits its applicability to other types of objects, such as pedestrians, bicyclists, or animals, which are also critical in real-world detection tasks. Furthermore, the effectiveness of the method proposed in this paper in other weather conditions (e.g., rain, fog, or night-time) has not been fully explored. Therefore, future work will focus on extending the Sec-CLOCs method to detect a wider range of objects, including pedestrians and other road users, as well as dealing with a variety of weather conditions other than snow, such as heavy rain, fog, and low visibility scenarios, which will further improve its robustness and adaptability.

## Figures and Tables

**Table 1:** YOLOv8s training parameters.

Model	Training Cycle	Batch Size	Image Size	Optimizer	Automatic Mixing Accuracy
YOLOv8s	300 epoch	16	640 × 640 pixels	SGD	True

**Table 2:** Training parameters for CLOCs.

Model	Training Cycle	Batch Size	Maximal Voxel	Worker Threads	Point Shuffling
CLOCs	160 epoch	1	16,000	3	True

**Table 3:** YOLOv8s experiments.

Model	Precision	Recall	mAP 50	mAP 50-95	F1 Score
YOLOv8s	90.7%	74.2%	83.5%	63.3%	81.5%
YOLOv8s+fasternet	89.8%	73.7%	83.6%	61.6%	81.0%
YOLOv8s+timm	89.8%	74.0%	84.0%	62.1%	81.0%
YOLOv8s+GhostHGNetV2	89.4%	74.7%	84.0%	63.6%	81.4%
YOLOv8s+SPPF-LSKA	91.8%	73.5%	83.8%	63.2%	81.6%
**YOLOv8s+DyHead **	**90.7%**	**74.8%**	**84.1%**	**64.3%**	**81.8%**
**YOLOv8s+Wise-IOU**	**91.3%**	**76.2%**	**85.7%**	**65.1%**	**83.1%**
**YOLOv8s+DyHead+Wise-IOU**	**90.1%**	**77.7%**	**86.0%**	**65.8%**	**83.4%**

**Table 4:** Sec-CLOCs ablation experiments.

Model	$A_{\mathrm{BEV}}$			$A_{3D}$		
	Easy	Moderate	Hard	Easy	Moderate	Hard
CLOCs	75.73%	66.76%	68.31%	47.92%	43.46%	45.57%
CLOCs+LIDROR	78.77%	70.56%	71.57%	52.72%	50.99%	52.28%
CLOCs+LIDROR+Improved YOLOv8s	83.60%	81.23%	80.78%	66.68%	64.17%	63.12%
**Our **	**84.50%**	**82.34%**	**81.76%**	**68.61%**	**64.69%**	**63.78%**

**Table 5:** Vehicle object detection results.

Model	$A_{\mathrm{BEV}}$			$A_{3D}$		
	Easy	Moderate	Hard	Easy	Moderate	Hard
AVOD	81.22%	71.76%	69.25%	68.55%	63.54%	56.68%
F-PointNet	81.56%	70.35%	67.25%	68.58%	64.59%	57.84%
F-ConvNet	80.68%	71.56%	69.11%	64.65%	57.45%	55.16%
PointRCNN	85.94%	82.31%	81.71%	70.11%	63.65%	62.68%
TANet	84.52%	82.23%	80.65%	67.46%	62.09%	60.66%
Snow-CLOCs	**86.21%**	79.81%	79.12%	**70.55%**	63.54%	62.83%
**Sec-CLOCs**	84.50%	**82.34%**	**81.76%**	68.61%	**64.69%**	**63.78%**

## Data Availability

The data used to support the results of this study are included in the article.

## Abbreviations

The following abbreviations are used in this manuscript:

TKLMR	Two-stage Knowledge Learning and Multi-contrastive Regularization
LIDROR	Low-Intensity Dynamic Radius Outlier Removal
